# Assessing the effectiveness of Interleukin-2 therapy in experimental type 1 diabetes

**DOI:** 10.1007/s12020-024-03753-z

**Published:** 2024-02-29

**Authors:** Zhengkang Luo, Mariela Mejia-Cordova, Nour Hamze, Elin Berggren, Saloni Chopra, Bilal Safi, Martin Blixt, Stellan Sandler, Kailash Singh

**Affiliations:** Department of Medical Cell Biology, Uppsala University, Uppsala, Sweden

**Keywords:** Type 1 diabetes (T1D), Interleukin-2 (IL-2), Regulatory B (Breg) cells, Regulatory T (Treg) cells, Streptozotocin

## Abstract

**Aim:**

Much focus of immunotherapy for type 1 diabetes (T1D) has been devoted on selectively boosting regulatory T (Treg) cells using low dose IL-2 due to their constitutive expression of IL-2Rα, CD25. However, several clinical trials using a low dose of IL-2 only showed a limited improvement of metabolic control. It can therefore be hypothesized that further decreasing IL-2 dosage may increase the selective responsiveness of Treg cells.

**Methods:**

We induced experimental T1D using multiple low dose streptozotocin (STZ) injections and treated the mice with an ultra-low dose IL-2 (uIL-2, approximately 7-fold lower than low dose). Immune response was studied using multicolor flow cytometry.

**Results:**

We found that uIL-2 did not protect STZ mice from developing hyperglycemia. It did neither increase Treg cell proportions, nor did it correct the phenotypic shift of Treg cells seen in T1D. It only partially decreased the proportion of IFN-γ^+^ T cells. Likewise, uIL-2 also did not protect the dysfunction of regulatory B (Breg) cells. Strikingly, when administered in combination with an anti-inflammatory cytokine IL-35, uIL-2 abrogated IL-35’s protective effect. Low dose IL-2, on the other hand, protected half of the STZ mice from developing hyperglycemia. No difference was found in the Treg and Breg response, and it only tended to decrease CD80 expression in macrophages and dendritic cells.

**Conclusion:**

In conclusion, further decreasing IL-2 dosage may not be a suitable approach for T1D therapy, and the limited success suggests that an alternative low dose IL-2 therapy strategy or other immunotherapies should be considered.

## Introduction

Type 1 diabetes (T1D) is an autoimmune disease, where pancreatic β cells are damaged by an unknown event [1]. It is hypothesized that the release of islet antigens results in an autoimmune attack to β cells and eventually deficiency of insulin production [2]. CD4^+^CD25^+^Foxp3^+^ regulatory T (Treg) cells are essential for immune tolerance and play an important role in preventing and reversing experimental T1D [3, 4].

IL-2 is a T cell stimulating cytokine, and Treg cells require IL-2 to maintain their suppressive function [5]. The lack of IL-2 is related to autoimmune diseases. For example, IL-2^−/−^ mice developed an inflammatory bowel disease [6]. The neutralization of IL-2 has been shown to accelerate the disease progression in NOD mice [7]. The IL-2 receptor (IL-2R) is composed of three chains: an α chain (IL-2Rα, CD25), a β chain (IL-2Rβ, CD122) and a γ chain (also known as the common γ chain, γc, CD132). When all the three chains are co-expressed on a cell, IL-2 is bound with high affinity [8]. The constitutive expression of CD25, and thus the high affinity IL-2 receptor on Treg cells makes low dose IL-2 an option to selectively boost Treg cell response.

Another type of regulatory immune cell is the regulatory B (Breg) cell. These cells are characterized as IL-10 and/or IL-35 producing B cells, but no Breg specific transcription marker has been identified. Breg cells have been shown to suppress autoimmune diseases in animal models [9–13]. We designate CD19^+^CD1d^+^CD5^+^ cells as Breg cells in the present study due to the complexity of Breg subsets and markers. In a previous study, we have shown an impaired function of Breg cells in an experimental model of T1D and patients with T1D [14].

The dosing of IL-2 is crucial in the attempt to prevent or reverse T1D. High dose IL-2 was initially used as a cancer immunotherapy [15]. A previous study showed that high dose IL-2 was toxic to NOD mice and accelerated T1D progression [16]. On the other hand, low dose IL-2 prevented diabetes in NOD mice and partially induced diabetes remission [17, 18]. Therefore, further decreasing the IL-2 dosage seemed to be a logical approach to improving the effect of IL-2 therapy in patients with T1D. Thus, we used ultra-low dose IL-2 (approximately 7-fold lower than low dose) treatment in the multiple low dose streptozotocin (STZ) mouse model of T1D and assessed its effectiveness in preventing the disease.

## Materials and methods

### Animals

Male CD-1 mice were obtained from Charles River (Hannover, Germany) and all the experiments were approved by the local animal ethical board (ethical approval code: C154/2015, 5.8.18-11099/2021). CD-1 mouse strain was used in this study because it has the highest response to STZ injections compared with other commonly used mouse strains [19].

Mice were divided into five treatment groups:

1. vehicle + PBS (vehicle) group,
2. MLDSTZ + PBS (STZ) group,
3. MLDSTZ + ultra-low dose IL-2 (uIL-2) group,
4. MLDSTZ + ultra-low dose IL-2 + IL-35 (uIL-2 + IL-35) group and
5. MLDSTZ + low dose IL-2 (ldIL-2) group.

Mice in the vehicle group were injected i.p. with saline for five consecutive days and after that with PBS for eight days. The other mice were first injected i.p. with STZ (Sigma, St Louise, MO, USA; 40 mg/kg body weight) dissolved in 200 µl saline solution for five consecutive days. Multiple low dose STZ injection was employed in this study because it has been shown to trigger insulitis [20], which is a hallmark of T1D. On the contrary, a single high dose of STZ can only cause an almost complete destruction of the β-cells [21], but mononuclear cell infiltration in the islet of Langerhans could only be found in 10% of the mice [22]. After the low dose STZ injections, the mice were injected i.p. with PBS or 200 µl PBS containing low dose IL-2 (Recombinant Mouse IL-2 Protein, #402-ML/CF, R&D Systems, Minneapolis, MN, USA; 20,000 IU/day, or 2 MIU/m^2^/day), ultra-low dose IL-2 (Mouse IL-2 Carrier-Free Recombinant Protein, #34-8021-82, eBioscience, San Diego, CA, USA; 2850 IU/day, or 0.285 MIU/m^2^/day), ultra-low dose IL-2 + IL-35 (recombinant mouse IL-35, Chimerigen, Liestal, Switzerland; 0.75 μg/day) for eight days. A timeline diagram of the treatments is shown in the Supplementary Fig. S1. Blood glucose levels were measured using a blood glucose meter (FreeStyle Freedom Lite^TM^). Mice with blood glucose higher than 11.1 mmol/L were considered diabetic. The experiment was performed twice with *n* = 2 or 3 for each group each time.

### Tissue preparation

Mice were sacrificed one day after the last injection of cytokine treatment. Pancreata were saved in formaldehyde and embedded in paraffin for histological analysis. Thymi, and spleens were squeezed with a pair of tweezers, and red blood cells were lysed with 0.2 M NH_4_Cl. The cells were then resuspended in Hanks’ balanced salt solution (Statens veterinärmedicinska anstalt, Uppsala, Sweden). Pancreatic draining lymph nodes (PDLNs) were grinded with a pair of tweezers on a sterile metal mesh and were then washed and resuspended with RPMI-1640 (Sigma-Aldrich) as described earlier [23, 24].

### Flow cytometry

The cell suspension was stained with the following surface antibodies; CD4 (RM4-5, eBioscience), CD8a (53-6.7, BD Biosciences, San Jose, CA, USA), CD25 (PC61, BioLegend, San Diego, CA, USA), CD19 (1D3, BD Biosciences), CD5 (53-7.3, BD Biosciences), CD1d (1B1, BioLegend), MHC-II (M5/114.15.2), CD11b (M1/70, BioLegend), CD11c (N418, BioLegend), F4/80 (BM8, BioLegend), PDCA-1 (927, BioLegend), XCR1 (ZET, BioLegend), CD172a (P84, BioLegend) and CD80 (16-10A1, BioLegend). They were then fixed and permeabilized with Fixation and Permeabilization Buffer (eBioscience) for 1 h and stained with intracellular antibodies overnight: Foxp3 (FJK-16s, eBioscience), Ebi3 (355022, R&D Systems), IL-12p35 (27537, R&D Systems), IFN-γ (XMG1.2, BioLegend) and IL-10 (JES5-16E3, BioLegend). Viability dye (Fixable viability Dye eFluor™ 780, eBioscience) was added prior to fixation, and Fc block (2.4G2, BD Biosciences) was added for both surface and intracellular staining. All the samples were analyzed using a BD LSR Fortessa at the BioVis platform (Uppsala University, Uppsala, Sweden). The flow cytometry data was analyzed by Flowlogic software (Inivai Technologies, Mentone, Australia) and FlowJo (Ashland, OR, USA). Gating strategies are shown in the Supplementary Fig. S2. Representative plots for IL-35 and IL-10 staining are previously shown [14]. Representative plots for IFN-γ and CD80 staining are shown in the Supplementary Figs. S3, 4.

### Histological analysis

Histological analysis was performed as previously described in detail to cover the sections from whole pancreas [25, 26]. In short, pancreata were saved in formaldehyde and embedded into paraffin blocks. Twelve slides (each containing 3-4 sections, and in between each section 25 µm tissue was discarded to avoid the same area for analysis) from each mouse were made using a microtome (Thermo Scientific Microm HM355S Rotary Microtome, Walldorf, Germany). They were then stained with guinea pig anti-insulin antibody (Fitzgerald, Acton, MA, USA) and peroxidase-conjugated anti-guinea pig antibody (Jackson ImmunoResearch Laboratories) in haematoxylin (Histolab products AB, Askim, Sweden). Three slides from each mouse (slide numbers 1, 6 and 12) were analyzed in a blind manner under a light microscope (Olympus BX53, Olympus Corporation, Tokyo, Japan) for scoring. Representative images are shown in the Supplementary Fig. S5. The extent of insulitis within the islets was assessed using the following grading system:

- Grade 0: Absence of immune cell infiltration
- Grade 1: Peri-infiltration
- Grade 2: Infiltration involving less than one-third (<1/3) of the islet
- Grade 3: Infiltration involving more than one-third (>1/3) of the islet
- Grade 4: Complete loss of islet structure.

### Statistical analysis

GraphPad Prism version 9.4.0 was used for all the statistical analysis. Repeated two-way ANOVA followed by Tukey’s test, one-way ANOVA followed by Tukey’s test, log-rank test followed by Bonferroni correction and Kruskal-Wallis test followed by Dunn’s test were performed to compare differences between groups.

## Results

### Ultra-low dose IL-2 does not prevent hyperglycemia in STZ mice

**Fig. 1:**
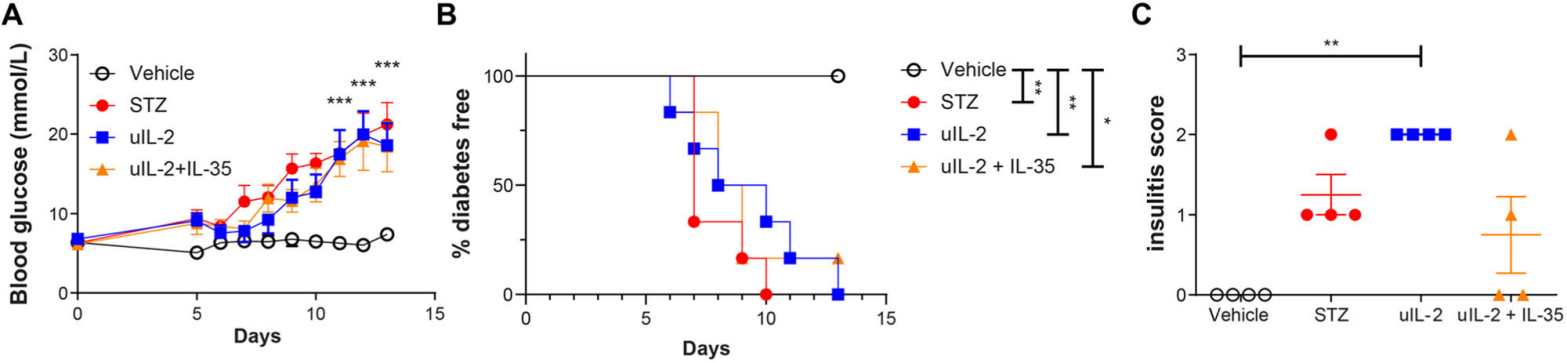
Ultra-low dose IL-2 does not prevent hyperglycemia in STZ mice. Male CD-1 mice received saline or STZ injection for 5 consecutive days, and PBS, uIL-2 or uIL-2 + IL-35 for the next 8 days. Blood glucose was monitored daily after day 5. **A** Blood glucose levels of mice. Repeated two-way ANOVA followed by Tukey’s test was performed, asterisks show the difference between Vehicle (*n* = 6) and STZ (*n* = 6), uIL-2 (*n* = 4), and uIL-2 + IL-35 (*n* = 6). **B** Percentage of diabetes free mice. Log-rank test followed by Bonferroni correction was performed. **C** Insulitis scores of the mice. Kruskal-Wallis test followed by Dunn’s test was used. Results are shown as mean ± SEM. The experiment was performed twice with *n* = 2 or 3 for each group each time. *, ** and *** denote *p* < 0.05, *p* < 0.01 and *p* < 0.001 respectively

Male CD-1 mice gradually developed hyperglycemia after STZ injections (Fig. 1A). However, uIL-2 did not prevent STZ mice from becoming hyperglycemic (Fig. 1B). Moreover, uIL-2 resulted in the highest insulitis scores of the mice (Fig. 1C). We have reported that IL-35 injections prevented STZ mice from becoming diabetic [14, 26, 27], we therefore used the combed injection of uIL-2 + IL-35. Surprisingly, uIL-2 abrogated the protective effect of IL-35 in preventing hyperglycemia and the majority of the mice still became diabetic (Fig. 1A–C, Supplementary Fig. S6). Thus, our data indicate that uIL-2 does not protect STZ mice from becoming diabetes.

### Ultra-low dose IL-2 does not prevent the phenotypic shift of Treg cells in thymic glands and in spleens of MLDSTZ mice

**Fig. 2:**
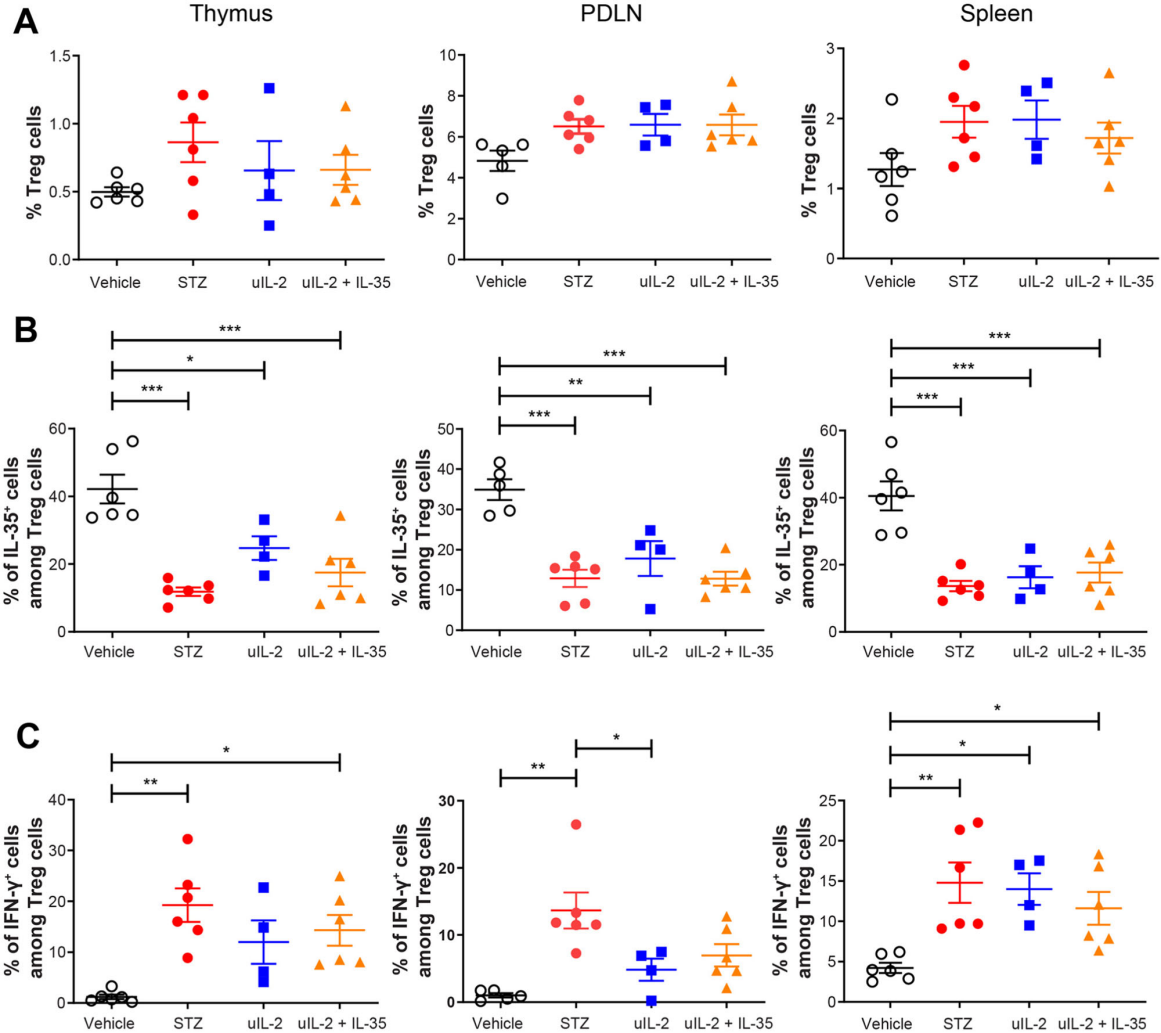
Ultra-low dose IL-2 does not prevent the phenotypic shift of Treg cells in STZ mice. Male CD-1 mice received saline or STZ injection for 5 consecutive days, and PBS, uIL-2 or uIL-2 + IL-35 for the next 8 days. **A** CD4^+^CD25^+^Foxp3^+^ Treg cell proportions. **B** Proportions of IL-35^+^ cells among Treg cells. **C** Proportions of IFN-γ^+^ cells among Treg cells. One-way ANOVA followed by Tukey’s test was performed. Results are shown as mean ± SEM. The experiment was performed twice with *n* = 2 or 3 for each group each time. *, ** and *** denote *p* < 0.05, *p* < 0.01 and *p* < 0.001 respectively

Treg cells have been reported to switch their phenotype and become dysfunctional in experimental T1D [26], producing less of the anti-inflammatory cytokine IL-35 and more proinflammatory cytokine IFN-γ [28]. Herein, we found that the treatments did not significantly increase the Treg cell proportion (Fig. 2A), nor did they prevent the phenotypic shift of Treg cells in STZ mice (Fig. 2B, C). However, treatment with uIL-2 lowered the percentage of IFN-γ^+^ cells among Treg cells in the PDLN compared with STZ mice (Fig. 2C). An increase in the Foxp3 MFI in CD4^+^ T cells was found in the spleen in uIL-2 treated mice compared with vehicle-treated mice, but no other difference in the Foxp3 MFI in CD4^+^ T cells or Treg cells was found (Supplementary Fig. S7A, B). Increased proportions of CD25^+^ cells among CD4^+^ T cells in the thymus, PDLN and spleen in STZ mice, and in the spleen in the uIL-2 treated mice (Supplementary Fig. S7C). There was no difference in the proportions of CD25^+^ cells among CD4^+^Foxp3^+^ cells (Supplementary Fig. S7D).

**Fig. 3:**
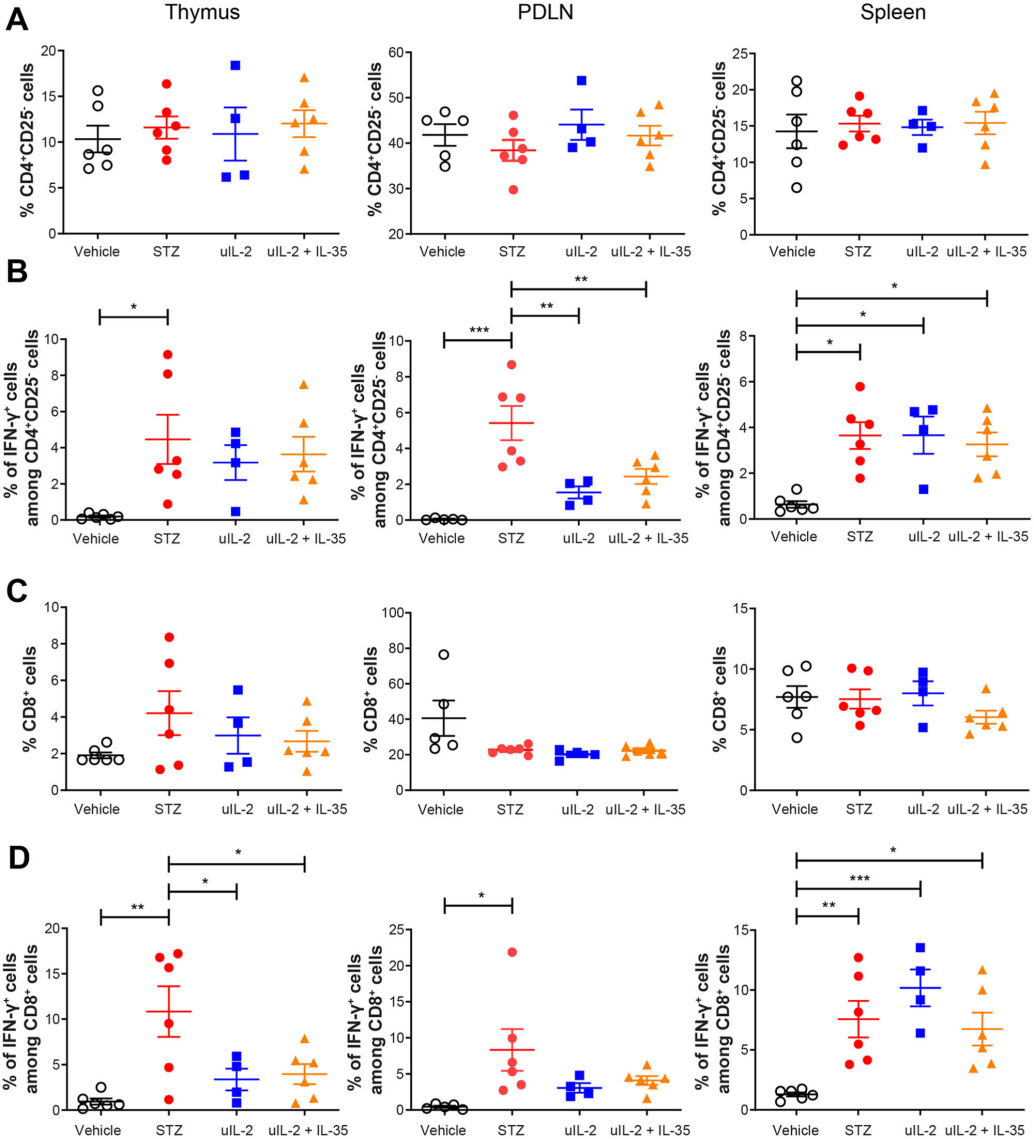
Ultra-low dose IL-2 partially lowers IFN-γ production in T cells. Male CD-1 mice received saline or STZ injection for 5 consecutive days, and PBS, uIL-2 or uIL-2 + IL-35 for the next 8 days. **A** Proportions of CD4^+^CD25^−^ T cells. **B** Proportions of IFN-γ^+^ cells among CD4^+^CD25^−^ T cells. **C** Proportions of CD8^+^ T cells. **D** Proportions of IFN-γ^+^ cells among CD8^+^ T cells. One-way ANOVA followed by Tukey’s test was performed. Results are shown as mean ± SEM. The experiment was performed twice with *n* = 2 or 3 for each group each time. *, ** and *** denote *p* < 0.05, *p* < 0.01 and *p* < 0.001 respectively

No difference was observed in the proportions of CD4^+^CD25^−^ and CD8^+^ T cells (Fig. 3A, C). uIL-2 and uIL-2 + IL-35 lowered the percentages of IFN-γ^+^ cells among CD4^+^CD25^−^ T cells in the PDLN, but did not do that in the spleen (Fig. 3B). Similarly, uIL-2 and uIL-2 + IL-35 lowered the percentages of IFN-γ^+^ cells among CD8^+^ T cells in the thymus but did not in the spleen (Fig. 3D). The percentages of IFN-γ^+^ cells among CD8^+^ T cells were higher in the spleen in treated groups than in the vehicle group (Fig. 3D). These data indicate that uIL-2 partially decreases IFN-γ production in T cells but does not protect the Treg cells’ phenotype in STZ mice.

### Ultra-low dose IL-2 does not prevent Breg cell dysfunction in STZ mice

**Fig. 4:**
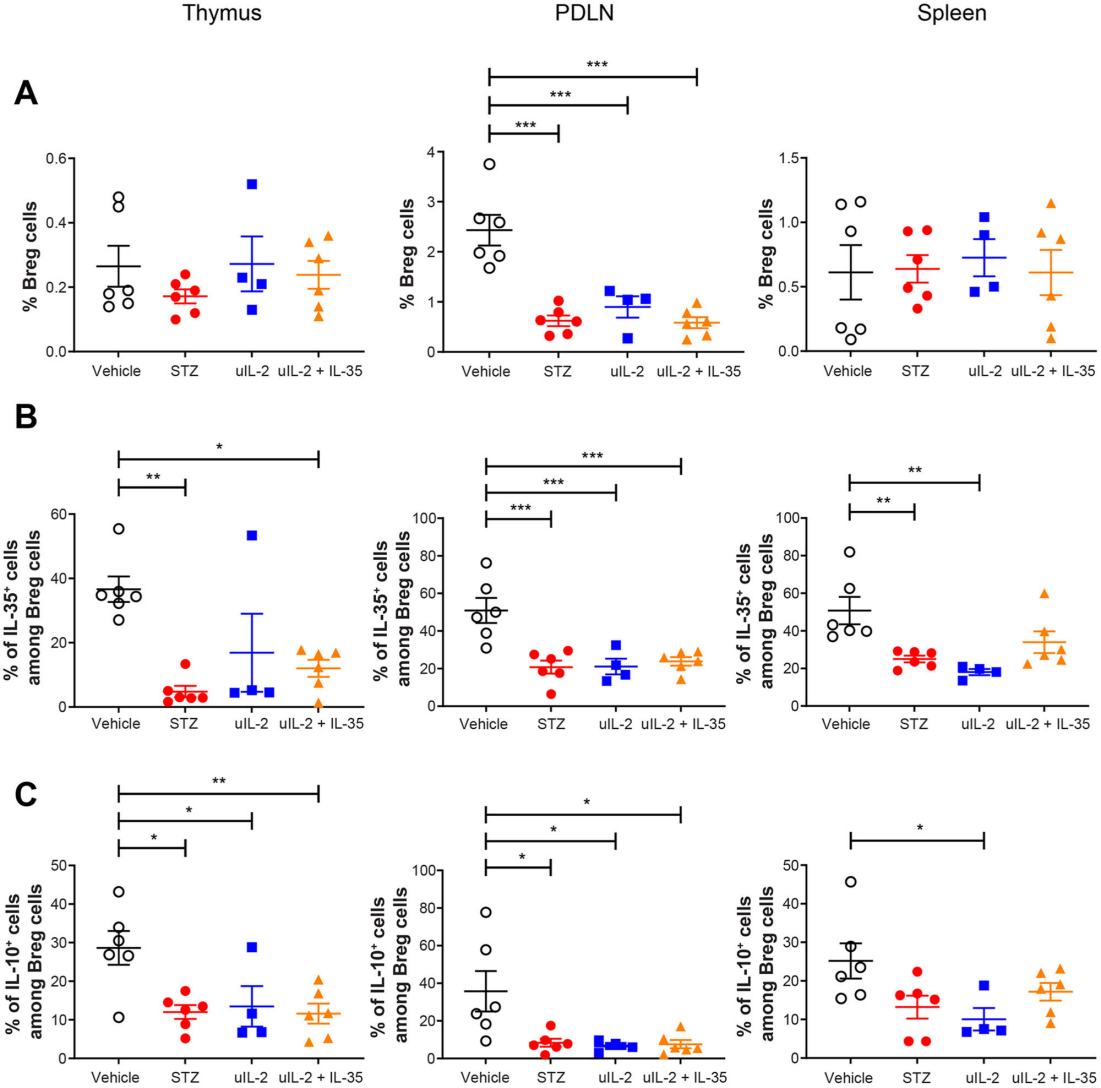
Ultra-low dose IL-2 does not prevent Breg cell dysfunction in STZ mice. Male CD-1 mice received saline or STZ injection for 5 consecutive days, and PBS, uIL-2 or uIL-2 + IL-35 for the next 8 days. **A** Proportions of CD19^+^CD1d^+^CD5^+^ Breg cells. **B** Proportions of IL-35^+^ cells among Breg cells. **C** Proportions of IL-10^+^ cells among Breg cells. One-way ANOVA followed by Tukey’s test was performed. Results are shown as mean ± SEM. The experiment was performed twice with *n* = 2 or 3 for each group each time. *, ** and *** denote *p* < 0.05, *p* < 0.01 and *p* < 0.001 respectively

Next, we investigated the Breg cell response after different treatments as previously we have shown that the response of Breg cells is impaired in T1D [14]. Consistent with our previous results, STZ treatment decreased the Breg cell proportion in the PDLN but did not in the thymus and spleen 13 days after the first injection (Fig. 4A) [14], and uIL-2 and uIL-2 + IL-35 treatment did not prevent this decrease in the PDLN (Fig. 4A). Similarly, uIL-2 did not prevent the decrease in IL-35 production in Breg cells in the PDLN and spleen, and uIL-2 + IL-35 did not in the thymus and PDLN (Fig. 4B). In addition, uIL-2 and uIL-2 + IL-35 did not prevent impaired IL-10 production from Breg cells after STZ injection (Fig. 4C). Therefore, these results showed that uIL-2 does not prevent the impairment of Breg cell function in STZ treated mice.

### Low dose IL-2 partially protects STZ mice from hyperglycemia

**Fig. 5:**
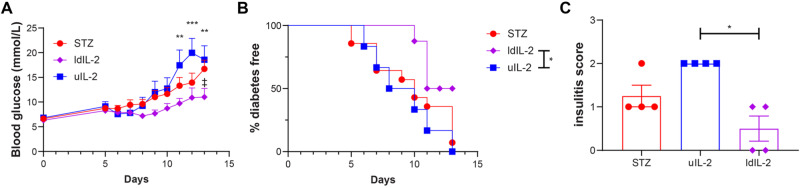
Low dose IL-2 partially protects STZ mice from hyperglycemia. Male CD-1 mice received saline or STZ injection for 5 consecutive days, and PBS, uIL-2 or ldIL-2 for the next 8 days. Blood glucose was monitored daily after day 5. **A** Blood glucose levels of mice. Repeated two-way ANOVA followed by Tukey’s test was performed, ** and *** denote *p* < 0.01 and *p* < 0.001 respectively between low dose IL-2 (ldIL-2, *n* = 8) and uIL-2 (*n* = 6), ‡ denotes *p* < 0.05 between ldIL-2 and STZ (*n* = 14). **B** Percentage of diabetes free mice. Log-rank test followed by Bonferroni correction was performed. * denotes *p* < 0.05. **C** Insulitis of the mice. Kruskal-Wallis test followed by Dunn’s test was used. * denotes *p* < 0.05. Results are shown as mean ± SEM

As the ultra-low dose IL-2 treatment did not show any protective effect against STZ, we increased the IL-2 dose and compared its effect in STZ mice. In contrast to the assumption that ultra-low dose IL-2 treatment causes a better glycemic control than low dose IL-2, we found that low dose IL-2 resulted in lower blood glucose levels than uIL-2 did (Fig. 5A), and only 50% of low dose IL-2 treated mice developed hyperglycemia compared with 100% in uIL-2 group (Fig. 5B). Low dose IL-2 treatment also improved the insulitis state compared with uIL-2 (Fig. 5C).

### Low dose IL-2 tends to reduce the response of antigen presenting cells

**Fig. 6:**
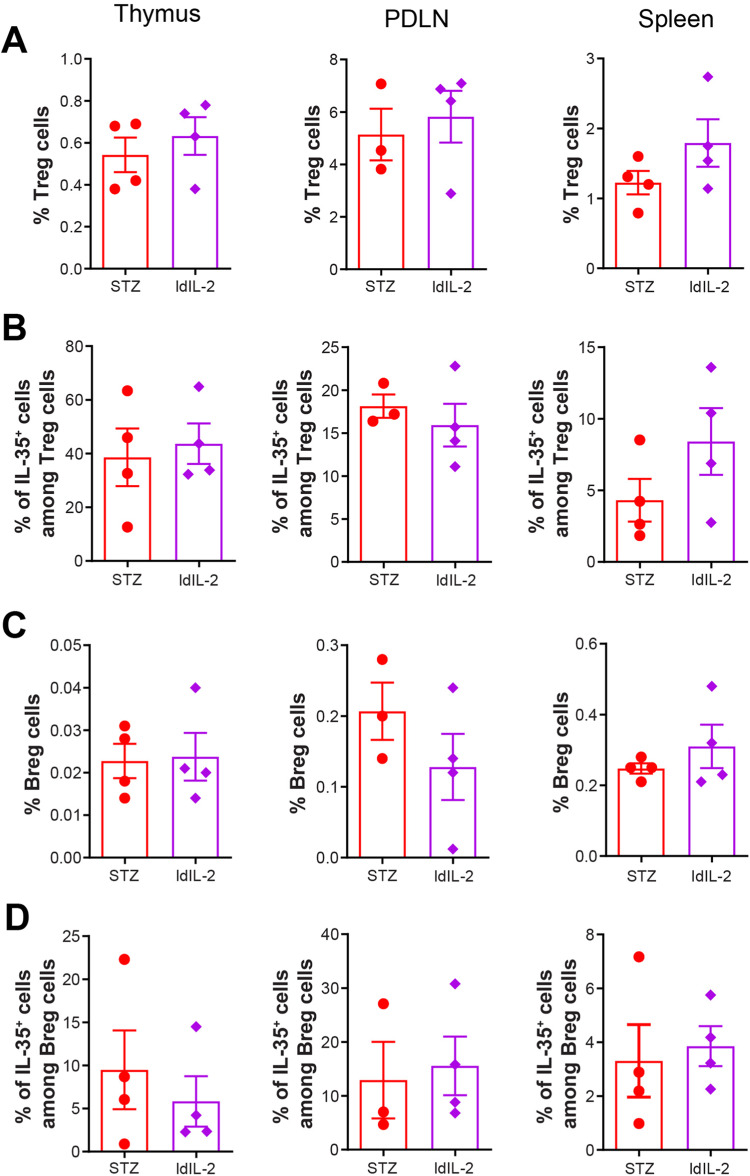
Low dose IL-2 does not change Treg and Breg cell phenotype. Male CD-1 mice received STZ injection for 5 consecutive days, and PBS or low dose IL-2 (ldIL-2) for the next 8 days. **A** Proportions of CD4^+^CD25^+^Foxp3^+^ Treg cells. **B** Proportions of IL-35^+^ cells among Treg cells. **C** Proportions of CD19^+^CD1d^+^CD5^+^ Breg cells. **D** Proportions of IL-35^+^ cells among Breg cells. Unpaired t test was performed. Results are shown as mean ± SEM. The experiment was performed twice with *n* = 2 for each group each time

Treg and Breg cell response was also studied after low dose IL-2 treatment. No difference in the proportions of Treg cells and IL-35^+^ cells among Treg cells was found in the thymus, PDLN and spleen (Fig. 6A, B). Low dose IL-2 did not increase Foxp3 MFI in CD4^+^ T cells or Treg cells, but it increased the proportions of CD25^+^ cells among CD4^+^ T cells in the spleen and among CD4^+^Foxp3^+^ cells in the thymus (Supplementary Fig. S8). No difference in CD8^+^ T cell proportions was found, as well as in IFN-γ MFI in CD8^+^ T cells and in Treg cells (Supplementary Fig. S9). Similarly, low dose IL-2 treatment did not change the proportions of Breg cells and IL-35^+^ cells among Breg cells (Fig. 6C, D). There was also no difference in IL-10 MFI in Breg cells (Supplementary Fig. S9).

**Fig. 7:**
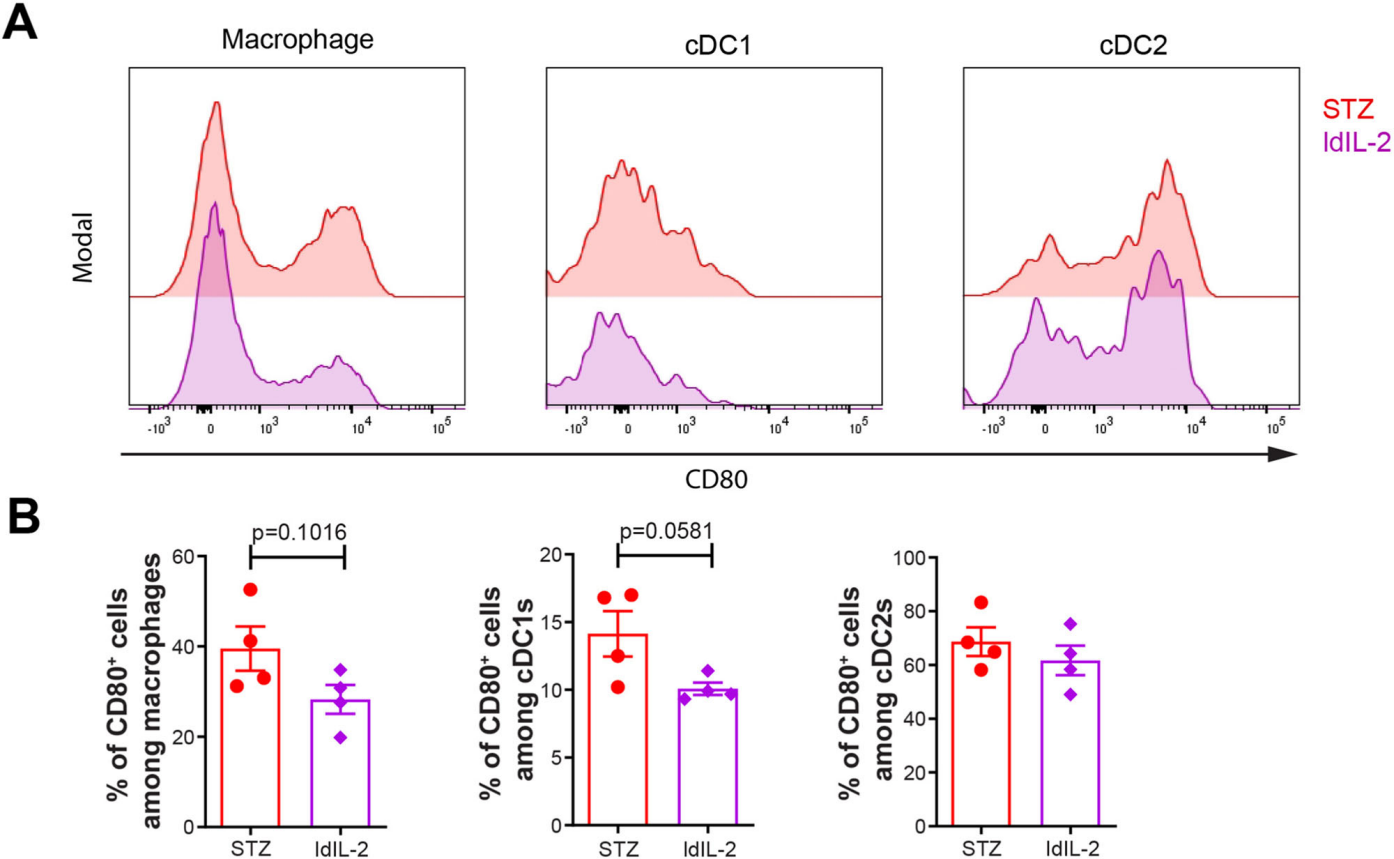
Low dose IL-2 tentatively decreases CD80 expression in antigen presenting cells. Male CD-1 mice received STZ injection for 5 consecutive days, and PBS or ldIL-2 for the next 8 days. Single splenocytes were stained and investigated using flow cytometry. **A** Fluorescence intensity of CD80 in CD11b^+^F4/80^+^ macrophages, MHC-II^+^CD11c^+^F4/80^-^XCR1^+^ cDC1s and MHC-II^+^CD11c^+^F4/80^-^CD172a^+^ cDC2s. **B** Proportions of CD80^+^ cells among macrophages, cDC1s and cDC2s. Unpaired *t*-test was performed. Results are shown as mean ± SEM. The experiment was performed twice with *n* = 2 for each group each time

As for antigen presenting cells, a slight decrease of CD80 fluorescence intensity was found in the macrophages, XCR1^+^ conventional dendritic cells type 1 (cDC1s) and CD172a^+^ conventional dendritic cells type 2 (cDC2s) after low dose IL-2 treatment (Fig. 7A). A non-significant decrease was found in the proportions of CD80^+^ cells among macrophages and cDC1s, but not among cDC2s (Fig. 7B). Altogether, our data illustrate that low dose IL-2 treatment did not alter Treg and Breg cell responses but reduced the response of antigen presenting cells, and could be the reason why low dose IL-2 only provided partial protection against diabetes.

Lastly, we directly compared the effects of uIL-2 and ldIL-2 on immune cell response (Supplementary Figs. S10–12) to further investigate the beneficial effect of ldIL-2 compared to uIL-2 for the prevention of T1D. Briefly, there was no difference in the proportion of Treg cells, but ldIL-2 caused lower CD4^+^CD25^−^ and CD8^+^ T cell proportions than uIL-2 in the spleen. Notably, ldIL-2 resulted in lower IFN-γ production Treg, CD4^+^CD25^−^ and CD8^+^ T cells than uIL-2 (Supplementary Figs. S10, S11). However, lower proportions of Breg cells were found in ldIL-2 than uIL-2, as well as IL-35 and IL-10 producing Breg cells in the spleen (Supplementary Fig. S12). However, due to the long gap between the time when those experiments were conducted, such comparison could only provide some insights for reference.

## Discussion

Earlier work in NOD mice has shown that low dose IL-2 has a partial protective effect against diabetes [18]. Another work on human peripheral blood mononuclear cells demonstrated that low dose IL-2 selectively activates Treg cells [29]. These studies support the use of low dose IL-2 as an immunotherapy in T1D. Therefore, much effort has been spent on low dose IL-2 treatment, and several clinical trials have been conducted [30–33]. As expected, low dose IL-2 induced the increase in Treg cell proportion in all these trials. However, a significant improvement of metabolic control was only observed in one trial [33], and improved maintenance of stimulated C-peptide was observed in another trial [31]. Studies by others and us also showed that the increased proportion of Treg cells fail to prevent the development of diabetes in T1D mouse models [17, 26]. Taken together, these studies suggest that low dose IL-2 treatment alone aiming for increasing Treg cell proportion may not be a very effective T1D therapy.

It should also be noted that the increase of Treg cell proportion or number does not necessarily protect against diabetes. A Treg cell dysfunction in T1D has been found in both human and mouse [34–36]. Our previous data showed that both Foxp3 mRNA levels and Treg cell proportions were elevated in the pancreas and spleen of STZ mice when diabetes was established [26]. Moreover, the improvement of Treg cell function by IL-35 was enough to prevent and reverse diabetes in STZ mice despite that Treg cell proportion did not increase. It is therefore probably of great importance that Treg cell function is improved for a successful treatment. Earlier work in NOD mice showed that low dose IL-2 improved Treg cell function in the islet but not in the systemics [18]. However, only a transient increase of CTLA-4 on Treg cells was found in one trial, and a transient increase of CTLA-4 and GITR in another [33, 37].

Finding an optimal IL-2 dose has been the focus of many studies [30, 31, 33, 38–43]. Indeed, high dose IL-2 was found to increase IFN-γ production in NK cells and CD8^+^ T cells, and to be lethal to NOD mice [16]. Thus, it should not be considered as a T1D therapy. Previous results and the current study showed that low dose IL-2 only partially protect mice from diabetes. One can therefore speculate that further decreasing the dose of IL-2 could provide better glucose control. However, we herein show that when IL-2 was administered at ultra-low dose (one-seventh of the dose shown to partially prevent T1D), IL-2’s protective effect could not be observed. Furthermore, neutralizing anti-IL-2 antibodies have been found in pre-diabetic and diabetic NOD mice and T1D patients [44], and they are more likely to disrupt IL-2’s protective effect when administered at a lower dose. Hence, further decreasing the dose of IL-2 may not be a suitable approach. Our current study showed that low dose IL-2 treatment protected 50% mice from diabetes after STZ injection, which is a promising result but is not optimal when considering other treatment like IL-35 provided almost complete protection in STZ mice [14, 26].

Since optimizing the IL-2 dose only had a limited success, a combination of low dose IL-2 with other treatments has been proposed. Low dose IL-2 together with an immunosuppressant rapamycin was found to prevent diabetes in NOD mice [45]. However, no metabolic benefit was found in a clinical trial [46], and a later study showed that rapamycin counteracts IL-2’s protective effect [16]. Another approach is combining low dose IL-2 with Treg cell adoptive transfer. A recent clinical trial showed that low dose IL-2 expanded transferred Treg cells but also expanded cytotoxic cells, with no improved islet metabolic function being observed [47]. Here, we also combined ultra-low dose IL-2 with IL-35, which has been shown to prevent and reverse T1D in mouse models. Much to our surprise, the addition of IL-2 abrogated IL-35’s protective effect. It is thus possible that increasing the IL-2 dose would not improve the outcome and that combining IL-2 with IL-35 might not be a successful therapy.

While the present study offers intriguing findings, it is essential to acknowledge several limitations that warrant consideration:

1. The current study does not delve into the mechanistic aspects concerning the impact of varying IL-2 doses, leaving gaps in our understanding.
2. Notably, the response of NK cells and ILCs remains unexplored, which could provide valuable insights into the observed effects.
3. The study’s restricted sample size is worth noting, as it may impact the generalizability and statistical robustness of the findings.
4. The properties and characterization of T cells, a crucial component, have regrettably not undergone comprehensive analysis.

Given these limitations, it becomes evident that further investigations are imperative to elucidate any immune-related mechanisms that might underlie the observed attenuation of IL-2’s protective efficacy within the STZ model.

In conclusion, our and others’ data demonstrate the complexity and difficulty in low dose IL-2 treatment, and suggest that an alternative low dose IL-2 therapy strategy or a combination with other immunotherapies could be considered.

### Supplementary information

Supplementary Figures
