# Supplementary material for: Assessing the effectiveness of Interleukin-2 therapy in experimental type 1 diabetes
Source: Endocrine. 2024 Feb 29;85(2):626–37. doi: 10.1007/s12020-024-03753-z (PMC11291609; doi:10.1007/s12020-024-03753-z)
Supplement: Supplementary file 1 — Supplementary Figures [file 12020_2024_3753_MOESM1_ESM.docx]

**Supplementary Figures**


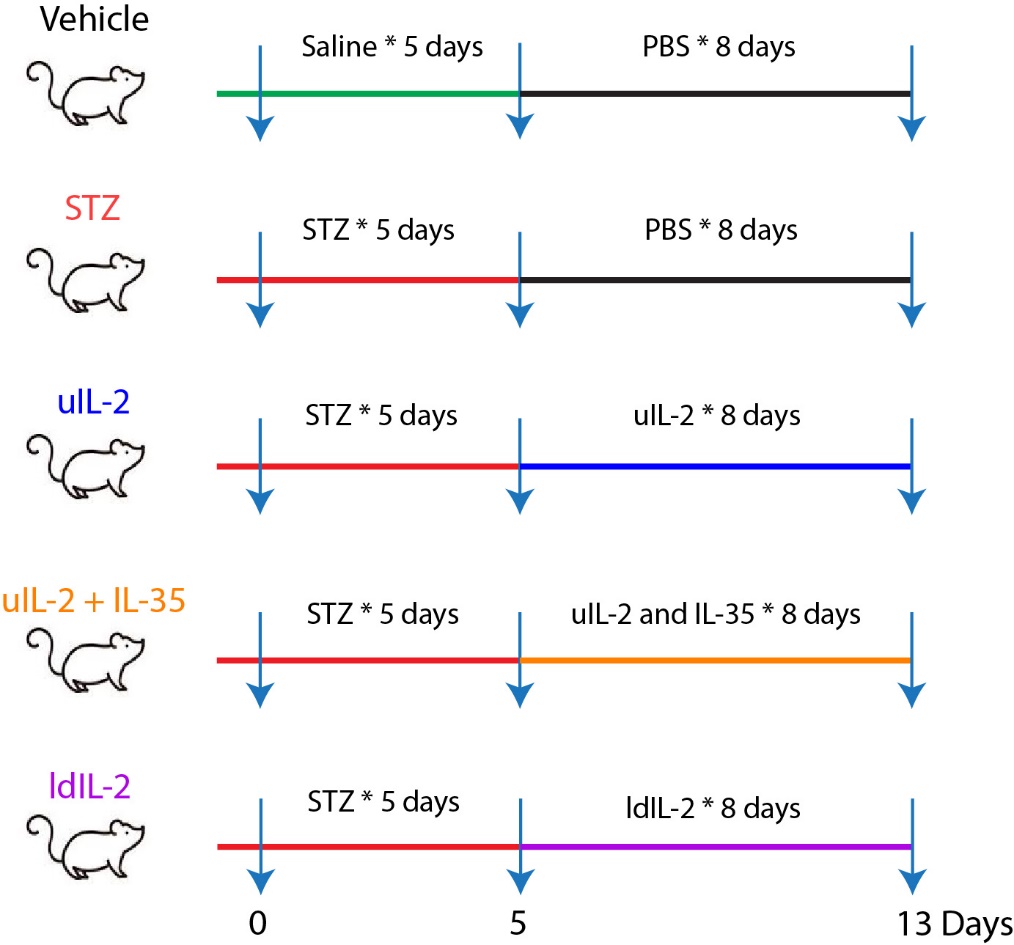


**Supplementary Figure S1.** A timeline diagram of the treatments. Male CD-1 mice received saline or low dose STZ injection for 5 days, and were injected with PBS or cytokines for the next 8 days. Blood glucose levels were monitored daily from day 5 after the first injection of STZ, and they were killed on day 13, after the first injection of STZ.


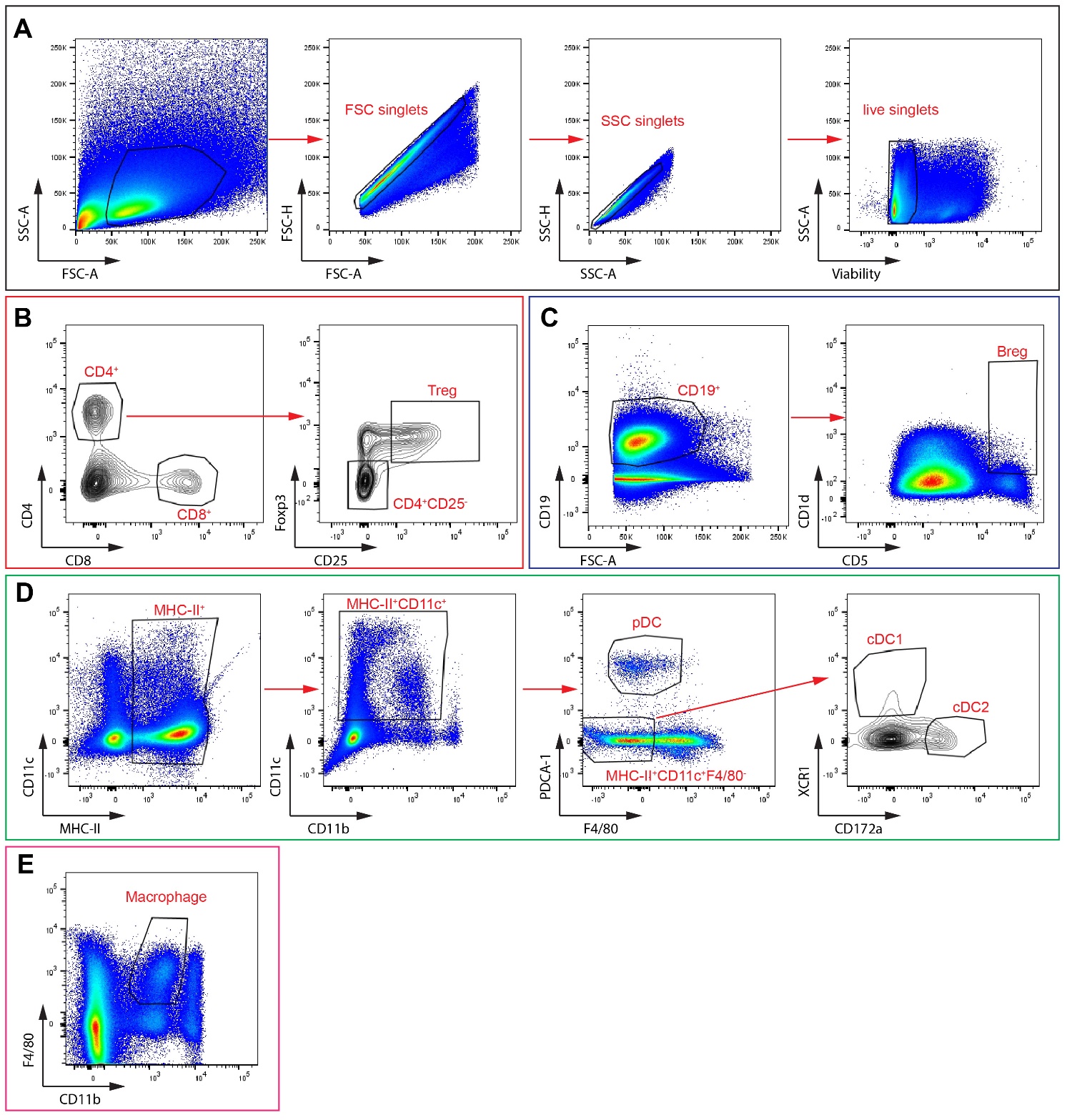


**Supplementary Figure S2.** Gating strategies for immune cells. Live singlets were selected based on FSC, SSC and viability staining (**A**). They were thereafter used for subsequent gatings for T cells and CD4^+^CD25^+^Foxp3^+^ Treg cells (**B**), B cells and CD19^+^CD1d^+^CD5^+^ Breg cells (**C**), MHC-II^+^CD11c^+^PDCA-1^+^ plasmacytoid dendritic cells (pDC), MHC-II^+^CD11c^+^F4/80^-^XCR1^+^ conventional dendritic cells type 1 (cDC1) and MHC-II^+^CD11c^+^F4/80^-^CD172a^+^ conventional dendritic cells type 2 (cDC2) (**D**), and CD11b^+^F4/80^+^ macrophages (**E**). The representative gatings were done on the spleen of a STZ mouse.


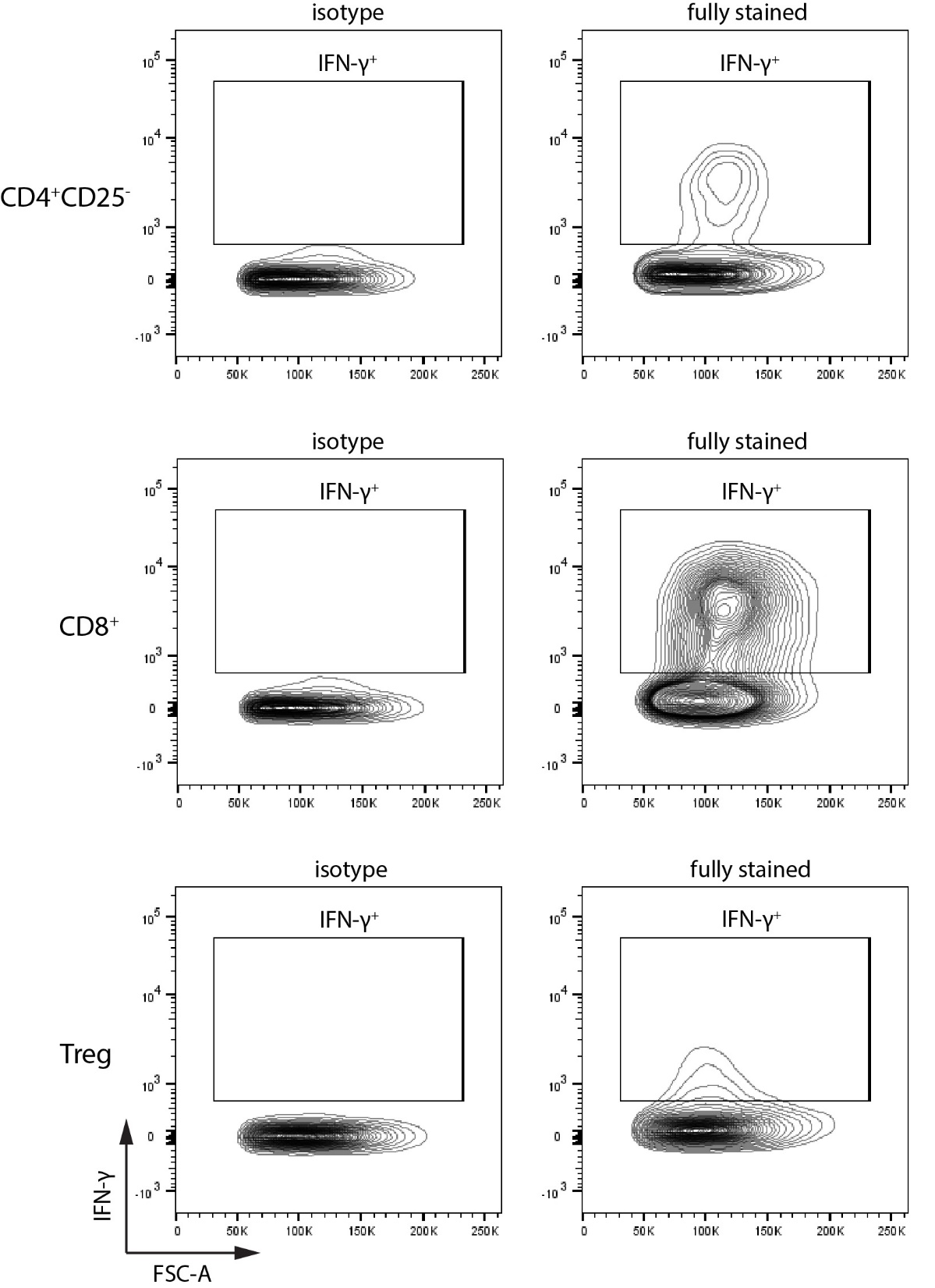


**Supplementary Figure S3.** Representative plots of IFN-γ staining of isotype or fully stained samples. Plots of gated CD4^+^CD25^-^ T cells (top), CD8^+^ T cells (middle) and Treg cells (bottom) are shown. The representative gatings were done on the spleen of a STZ mouse.


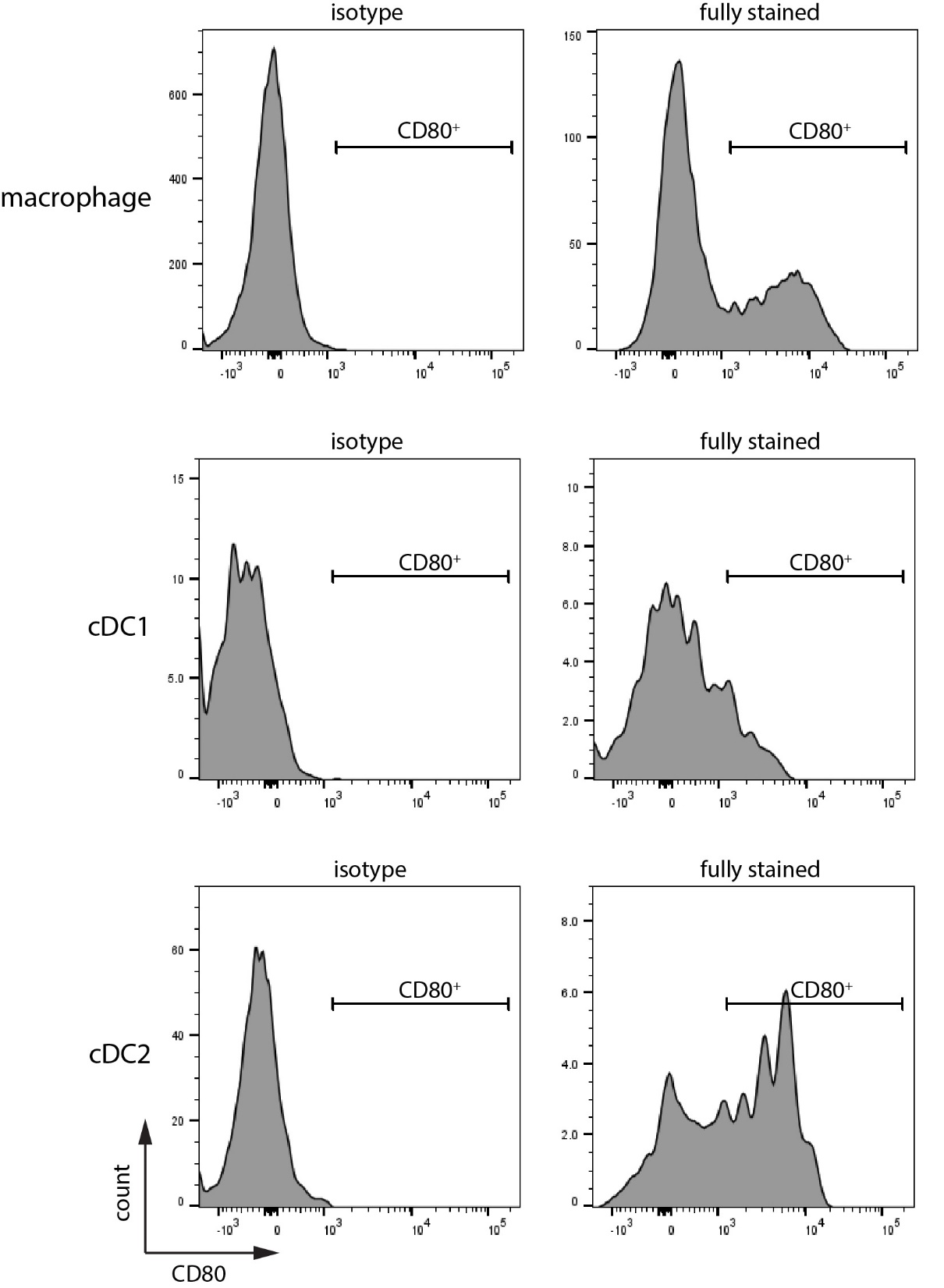


**Supplementary Figure S4.** Representative plots of CD80 staining of isotype or fully stained samples. Plots of gated macrophages (top), cCD1s (middle) and cDC2s (bottom) are shown. The representative gatings were done on the spleen of a STZ mouse.


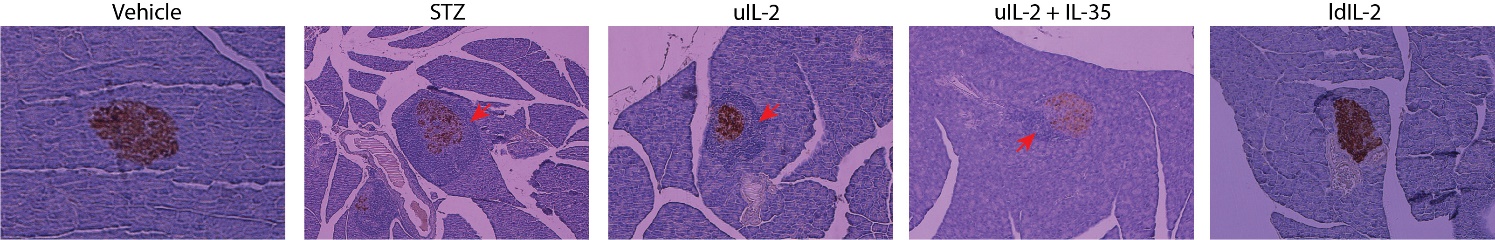


**Supplementary Figure S5.** Representative images of insulitis. Brown color indicates staining for insulin. Red arrows point to immune cell infiltration in the islet.


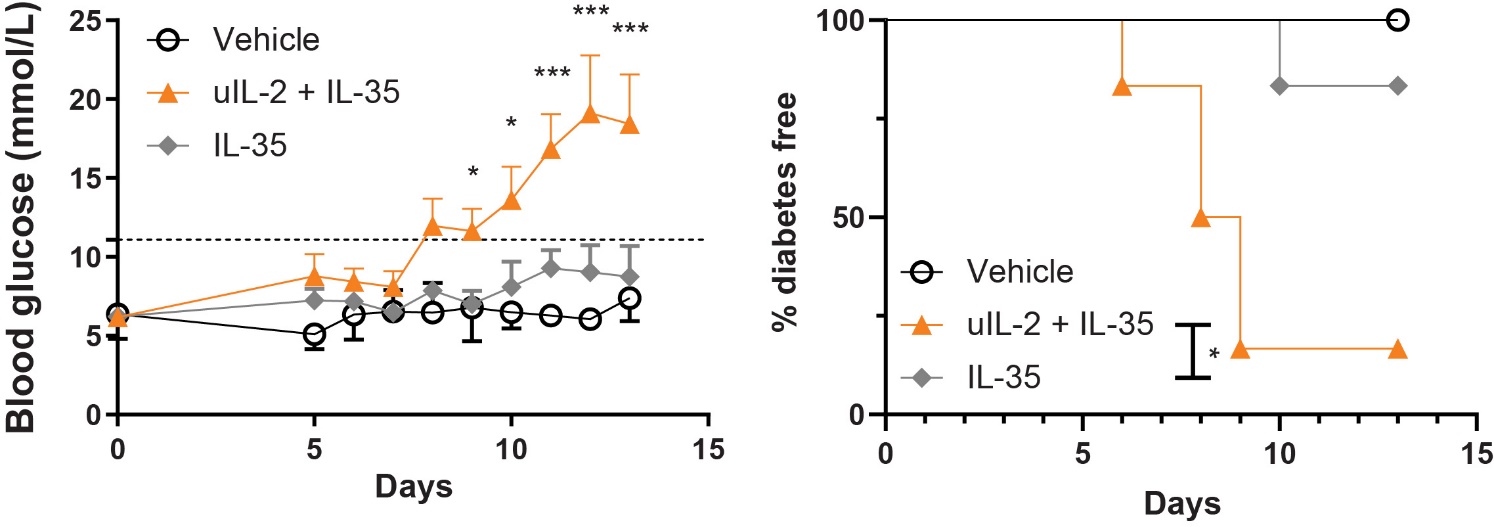


**Supplementary Figure S6.** Comparison of the preventing hyperglycemia between uIL-2 + IL-35 and IL-35. Male CD-1 mice were injected with STZ or saline for 5 consecutive days and injected daily with uIL-2 + IL-35, IL-35 and PBS for the next 8 days. Blood glucose was measured daily (left panel). Repeated measurement two-way ANOVA followed by Tukey’s test was performed, asterisks show the difference between uIL-2 + IL-35 and IL-35. Mice with blood glucose higher than 11.1 mmol/L were considered diabetic, and the percentages of diabetes free mice are shown (right panel). Log-rank test followed by Bonferroni correction was performed. Results are shown as mean ± SEM. * and *** denote p<0.05 and p<0.001 respectively. The IL-35 treatment experiment was performed by us at the same time as other treatment groups, and the blood glucose data of vehicle and IL-35 groups were previously published in reference (14). They were thereafter re-analyzed and compared with other treatment groups. (n=6)


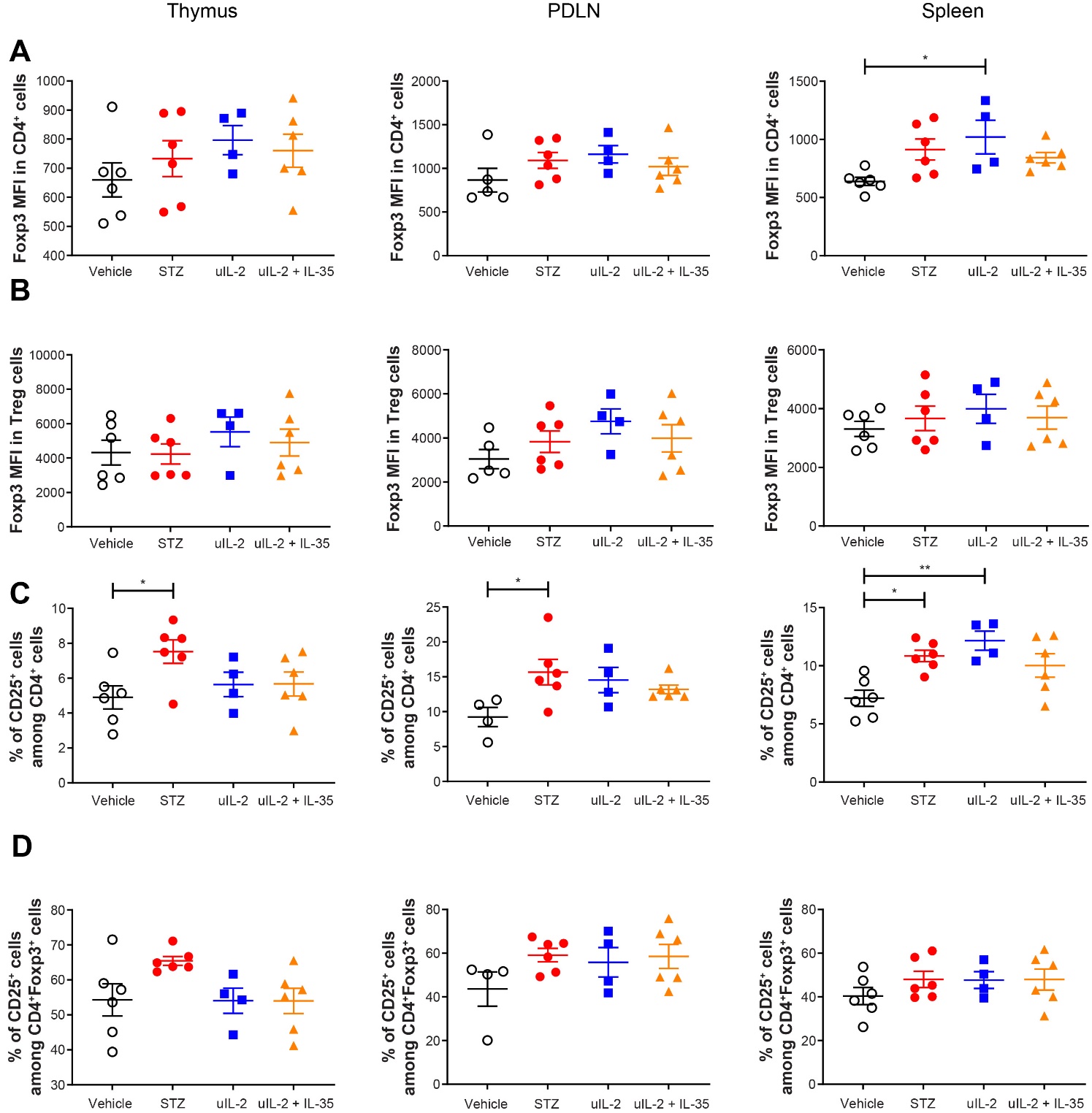


**Supplementary Figure S7.** Foxp3 MFI and CD25^+^ cell proportions. Foxp3 MFI in CD4^+^ T cells (A) and Treg cells (B), the proportions of CD25^+^ cells among CD4^+^ T cells (C) and CD4^+^Foxp3^+^ cells (D) are shown. One-way ANOVA followed by Tukey’s test was performed. Results are shown as mean ± SEM. * and ** denote p<0.05 and p<0.01 respectively.


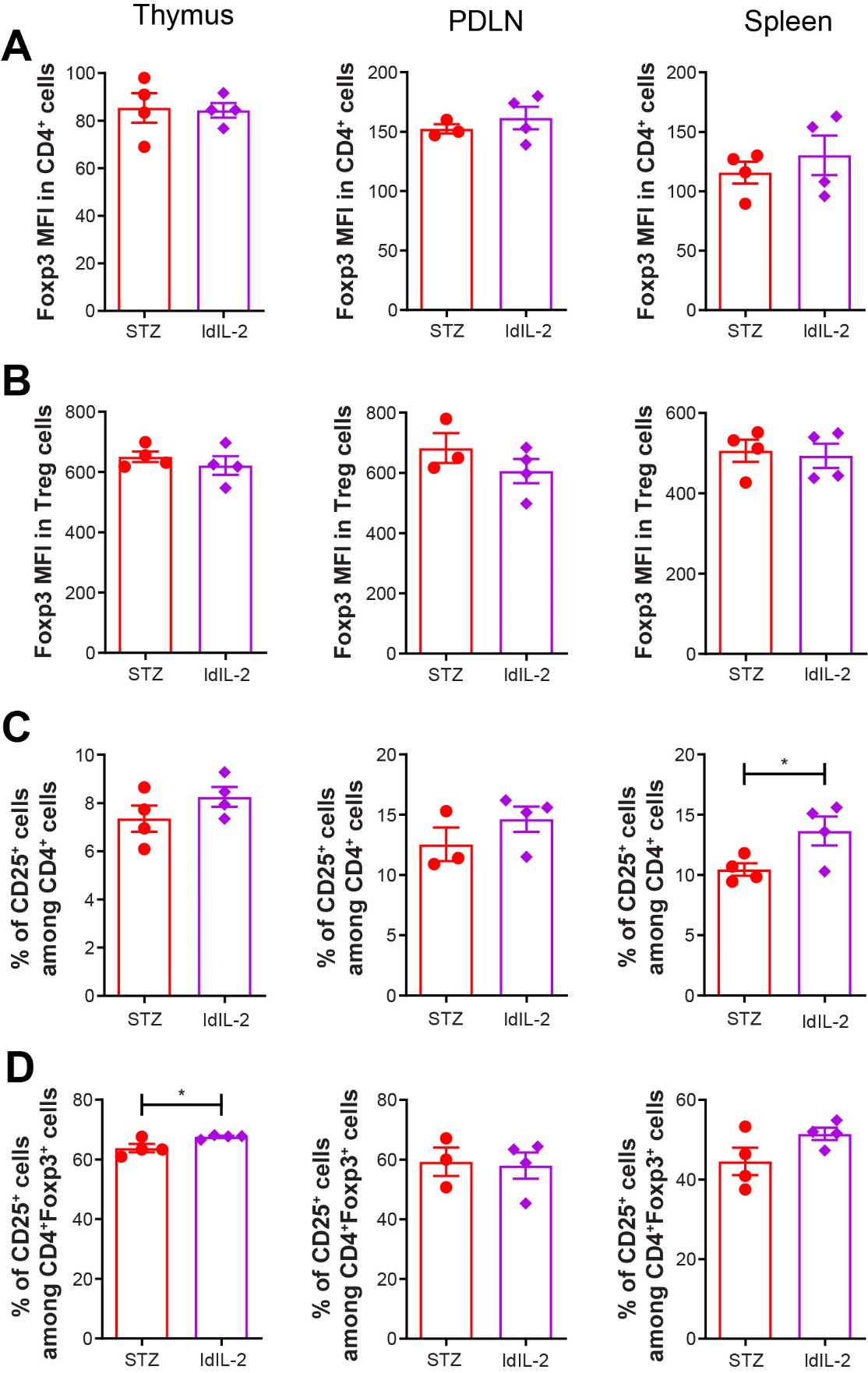


**Supplementary Figure S8.** ldIL-2 partially increases CD25^+^ cell proportions. Foxp3 MFI in CD4^+^ T cells (A) and Treg cells (B), the proportions of CD25^+^ cells among CD4^+^ T cells (C) and CD4^+^Foxp3^+^ cells (D) are shown. Unpaired t test was performed. Results are shown as mean ± SEM. * denotes p<0.05.


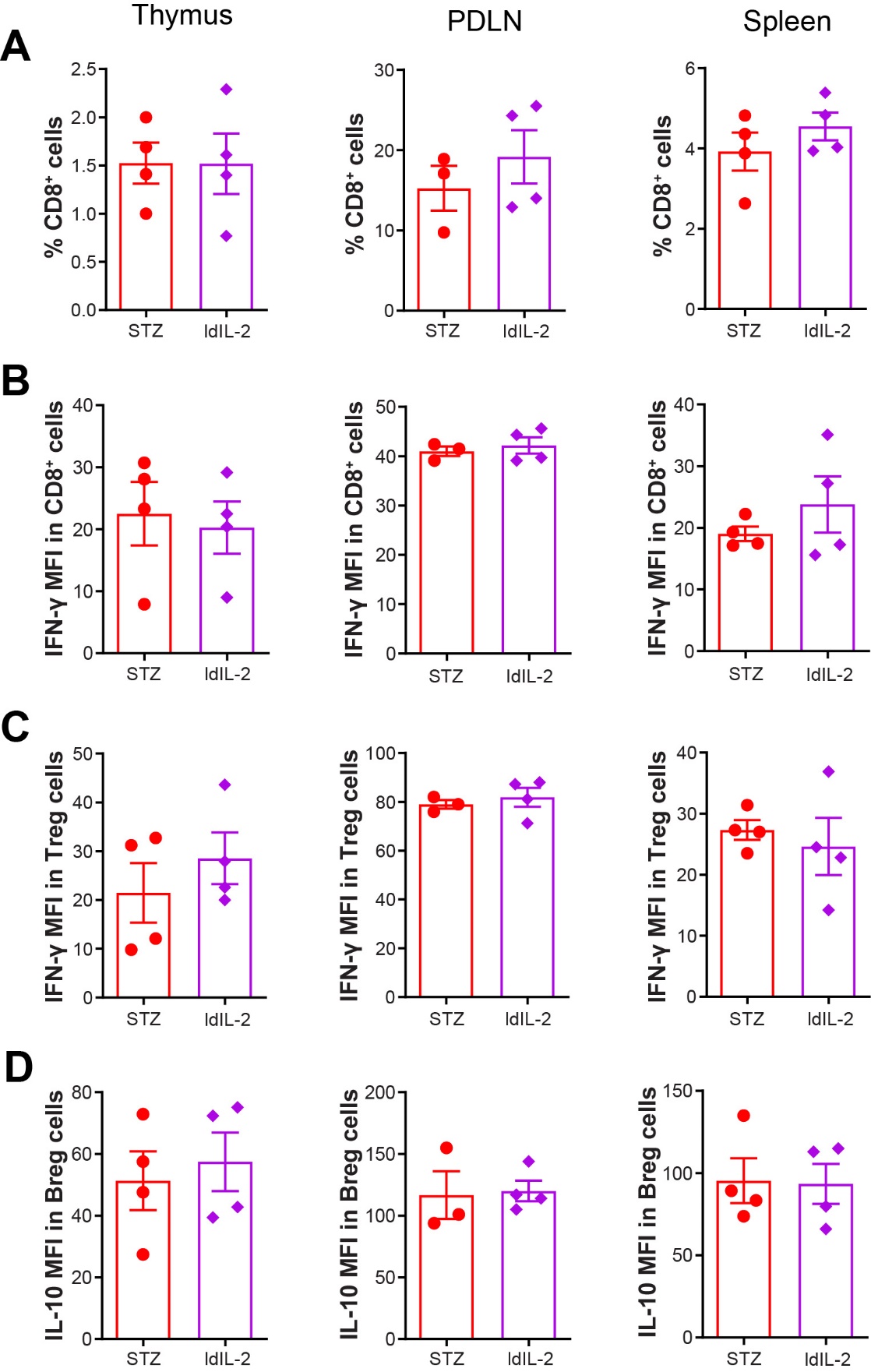


**Supplementary Figure S9.** ldIL-2 does not change the phenotype of CD8^+^ T cells, Treg cells and Breg cells. No difference was seen in the thymus, PDLN and spleen in the proportions of CD8^+^ T cells (A), IFN-γ MFI in CD8^+^ T cells (B) and Treg cells (C), and IL-10 MFI in Breg cells (D).


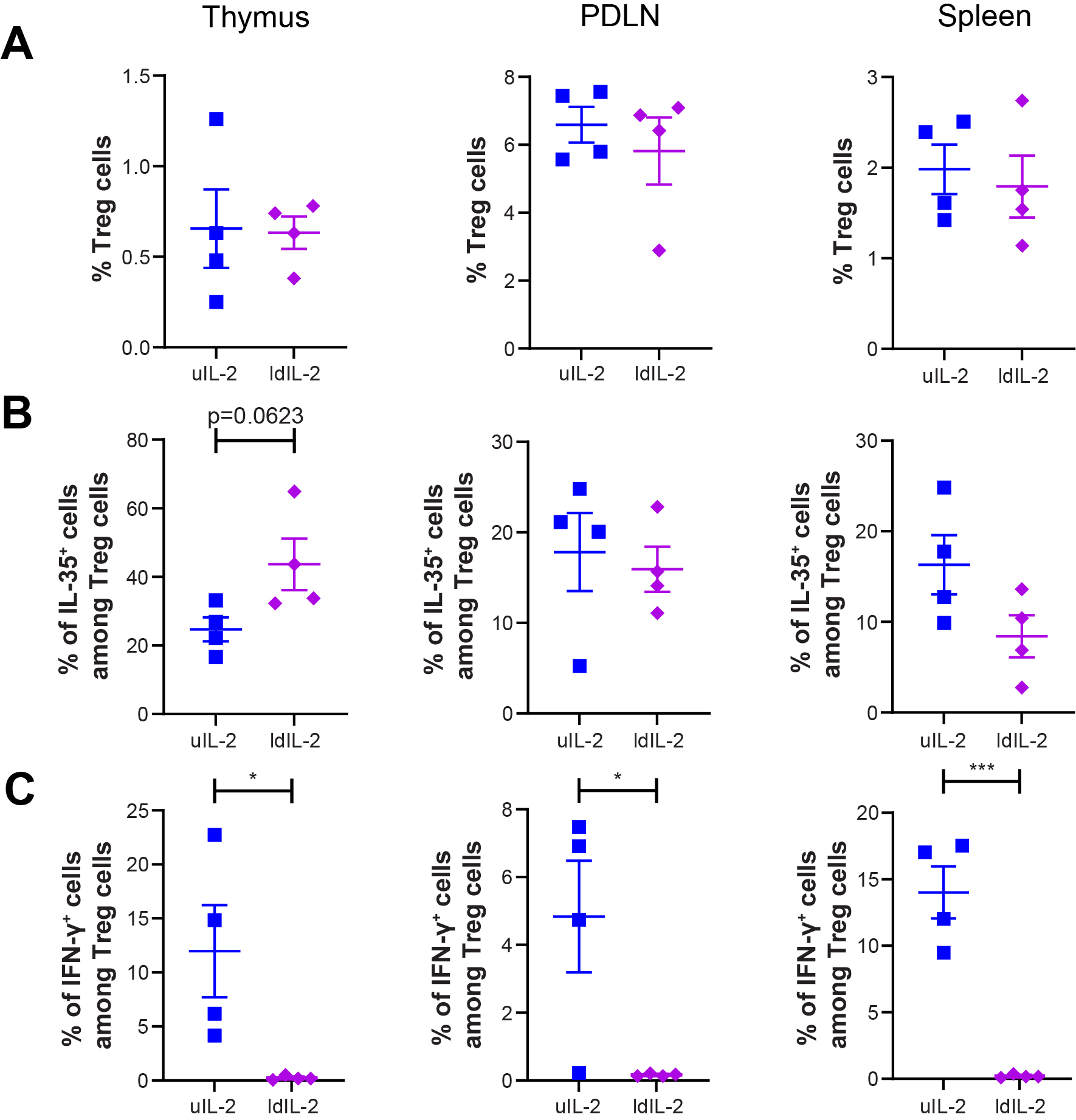


**Supplementary Figure S10.** ldIL-2 resulted in lower IFN-γ production in Treg cells than uIL-2. (A) The proportions of Treg cells. (B) The proportions of IL-35^+^ Treg cells. (C) The proportions of IFN-γ^+^ Treg cells.


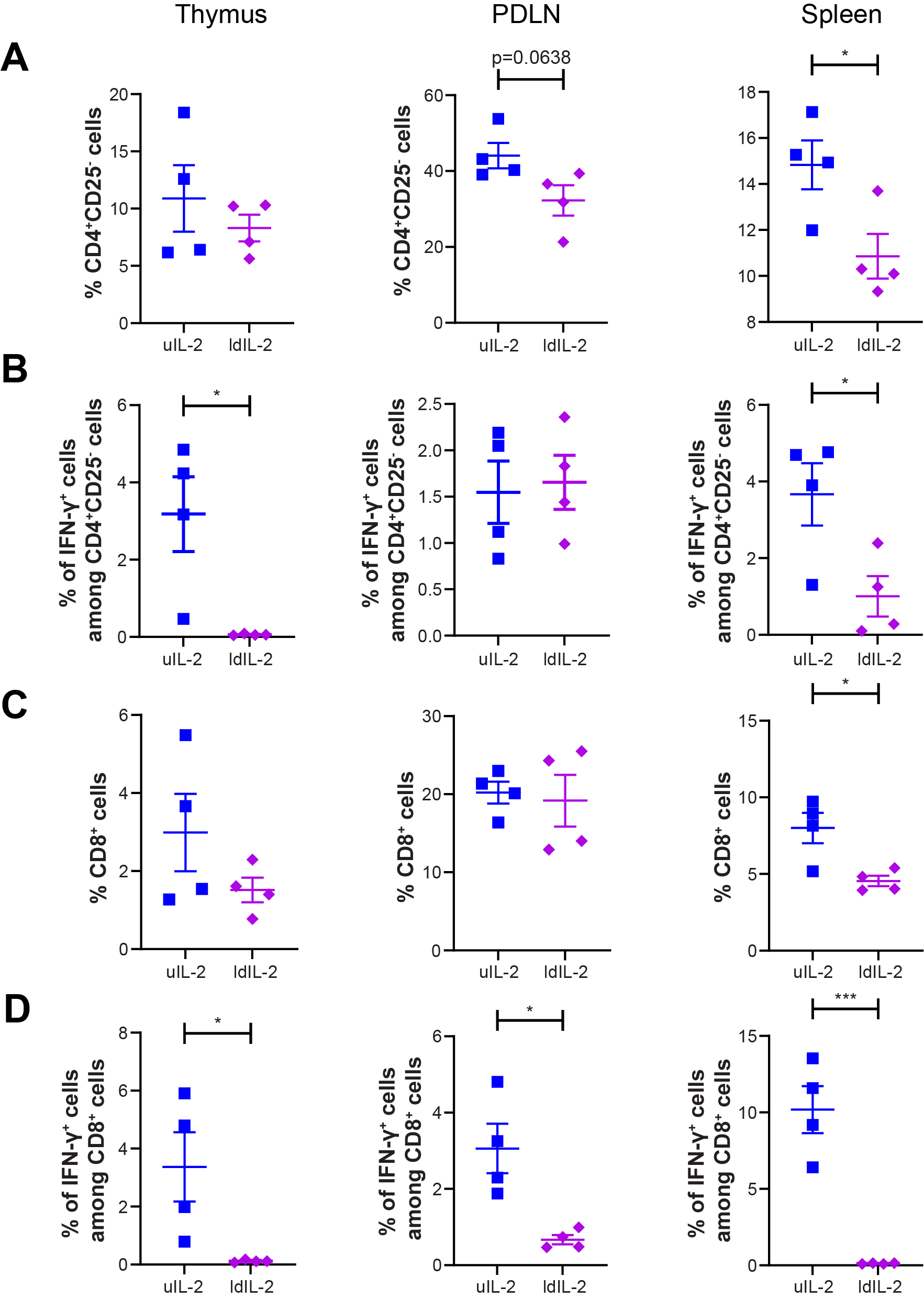


**Supplementary Figure S11.** ldIL-2 resulted in lower IFN-γ production in CD4^+^CD25^-^ and CD8^+^ T cells than uIL-2. (A) The proportions of CD4^+^CD25^-^ cells. (B) The proportions of IFN-γ^+^ cells among CD4^+^CD25^-^ cells. (C) The proportions of CD8^+^ cells. (D) The proportions of IFN-γ^+^ cells among CD8^+^ cells.


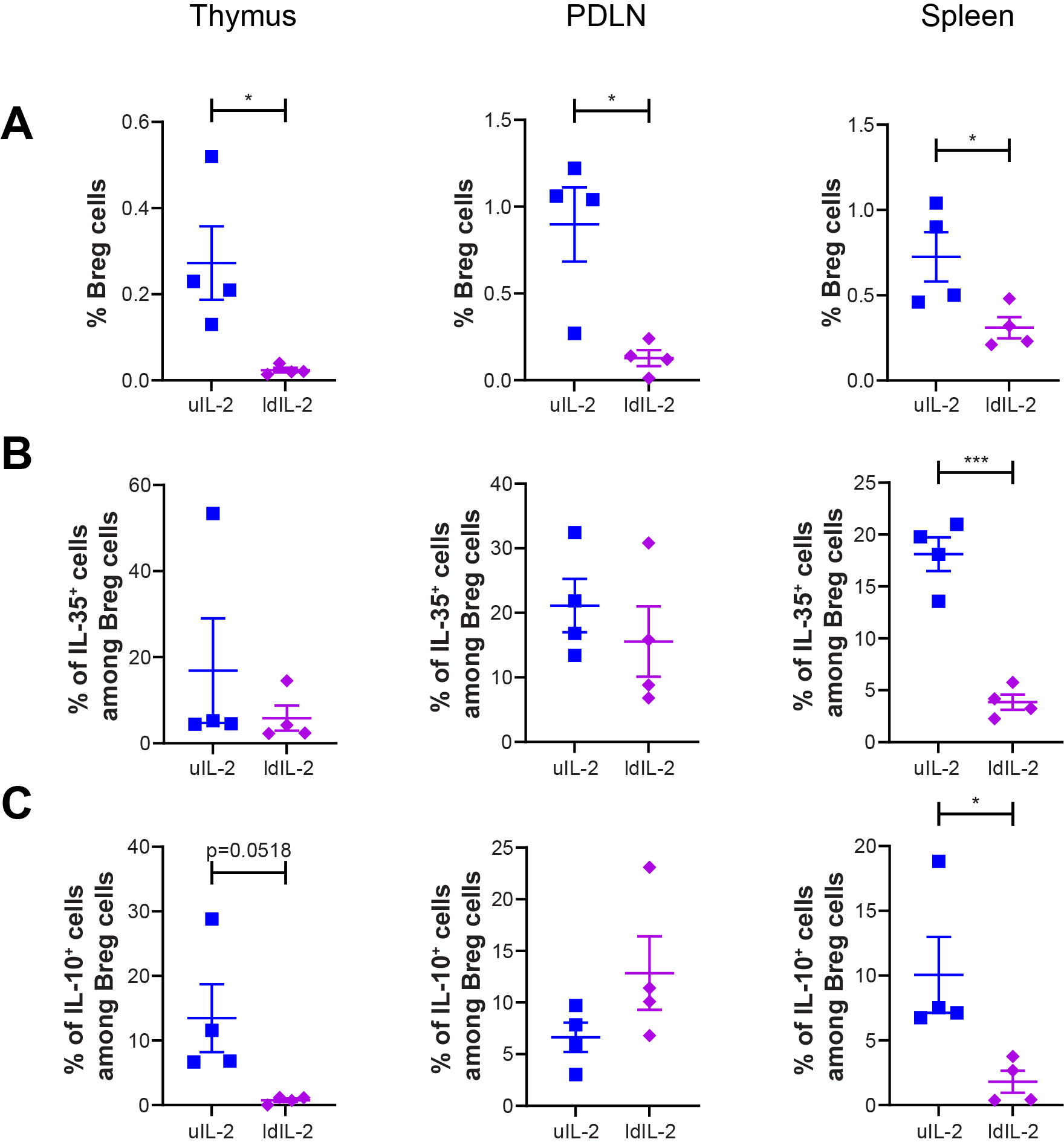


**Supplementary Figure S12.** ldIL-2 resulted in lower Breg cell proportions than uIL-2. (A) The proportions of Breg cells. (B) The proportions of IL-35^+^ cells among Breg cells. (C) The proportions of IL-10^+^ cells among Breg cells.
